# Quality-assured training in the evaluation of cochlear implant electrode position: a prospective experimental study

**DOI:** 10.1186/s12909-022-03464-x

**Published:** 2022-05-20

**Authors:** Alexander Mewes, Sebastian Burg, Goetz Brademann, Jan Andreas Dambon, Matthias Hey

**Affiliations:** 1 Universitätsklinikum Schleswig-Holstein (UKSH), Campus Kiel, Department of Otorhinolaryngology, Head and Neck Surgery, Kiel, Germany; 2 Christian-Albrechts-Universität (CAU) zu Kiel, Faculty of Medicine, Kiel, Germany

**Keywords:** Cochlear implant, Quality assurance, Electrode position, Electrode-to-modiolus distance, Angular depth of insertion

## Abstract

**Background:**

The objective of this study was to demonstrate the utility of an approach in training predoctoral medical students, to enable them to measure electrode-to-modiolus distances (EMDs) and insertion-depth angles (aDOIs) in cochlear implant (CI) imaging at the performance level of a single senior rater.

**Methods:**

This prospective experimental study was conducted on a clinical training dataset comprising patients undergoing cochlear implantation with a Nucleus® CI532 Slim Modiolar electrode (*N* = 20) or a CI512 Contour Advance electrode (*N* = 10). To assess the learning curves of a single medical student in measuring EMD and aDOI, interrater differences (senior–student) were compared with the intrarater differences of a single senior rater (test–retest). The interrater and intrarater range were both calculated as the distance between the 0.1th and 99.9th percentiles. A “deliberate practice” training approach was used to teach knowledge and skills, while correctives were applied to minimize faulty data-gathering and data synthesis.

**Results:**

Intrarater differences of the senior rater ranged from − 0.5 to 0.5 mm for EMD and − 14° to 16° for aDOI (respective medians: 0 mm and 0°). Use of the training approach led to interrater differences that matched this after the 4th (EMD) and 3rd (aDOI) feedback/measurement series had been provided to the student.

**Conclusions:**

The training approach enabled the student to evaluate the CI electrode position at the performance level of a senior rater. This finding may offer a basis for ongoing clinical quality assurance for the assessment of CI electrode position.

**Supplementary Information:**

The online version contains supplementary material available at 10.1186/s12909-022-03464-x.

## Background

Intracochlear positioning of the cochlear implant (CI) electrode is essential for successful placement of the CI adjacent to the modiolus with minimum intracochlear trauma. The electrode’s position is determined post-operatively, routinely by radiographic imaging techniques such as computer tomography (CT) or digital volume tomography (DVT). Therefore, measurements of electrode-to-modiolus distance (EMD) and angular depth of insertion (aDOI) are of clinical interest.

Within the framework of university education, these parameters are also measured by predoctoral medical students, and their inexperience may lead to diagnostic errors (missing findings or misinterpretation of findings) [1, 2]. Diagnostic errors are due primarily to cognitive bias, sources of which are, in radiology, usually associated with problems of visual perception (scanning, recognition or interpretation) [1, 3]. According to Graber et al. (2018) [3], sources of cognitive bias include inadequate knowledge or skill on the part of the rater, faulty data-gathering (i.e., gathering and measuring information on relevant variables) and faulty information synthesis (i.e., processing and verification) [1, 3]. In medicine, the most common sources of bias are linked to poor information synthesis, and this can be subdivided into further factors [3, 4].

The weight of these cognitive factors depends on the expertise of the rater. While experienced raters are especially vulnerable to drawing premature conclusions (fast or type 1 thinking) [5], the major problems in decision-making by medical students are inadequate knowledge and skills, faulty context generation, faulty triggering, misidentification and premature conclusions [4]. However, an inexperienced rater will have a more analytical approach to decision-making, even though this (type 2) thinking needs more time. Type 2 thinking by medical students can thus provide a basis for careful CI image evaluation. For this, the student must be given the specific knowledge and skills on the one hand, while correctives are applied to minimize faulty data collection and information synthesis on the other.

Therefore, the aim of this work was (1) to use “deliberate training” (in the sense of Ericsson et al. [6, 7]), to prepare a single student for this task while also reducing cognitive bias; (2) to analyze the student’s learning curves in measuring EMD and aDOI.

Our results lead us to hypothesize that it is possible to train a student such that he/she can measure EMD and aDOI at the level of an experienced senior rater.

## Methods

### Training approach

#### Deliberate practice to improve knowledge and skills

We assumed that the student had sufficient basic knowledge about the anatomy of the ear while still lacking specific skills, i.e., techniques for evaluating the intracochlear electrode position, software handling and knowledge about the influence of image-processing on the measurement results (especially effects of contrast enhancement and filtering). To teach these skills and bring the student up to the target performance level of an experienced senior rater, “deliberate practice” as defined by Ericsson et al. [6, 7] was conducted, including adequate access to training resources, a well-qualified trainer, learning goals to be achieved by the student, immediate feedback from the trainer and repeated fresh attempts by the student to achieve the goals gradually [6, 7].

#### Correctives to reduce faulty data-gathering and faulty information synthesis

As mentioned above, faulty context generation is a principal factor in diagnostic errors by medical students. However, decision-making by students may be affected by other cognitive biases, as noted in the background section. Therefore, our training approach addresses the following most common factors in information synthesis performed by medical students [4]. To reduce such bias, the following correctives, based on the taxonomy of Graber et al. (2018) [3], were implemented.

##### Faulty data-gathering

- Structured gathering of valid position parameters to describe the intracochlear electrode position (EMD, aDOI);
- Use of a consensual universal co-ordinate system of the cochlea to allow comparisons between raters.

##### Faulty information synthesis

- Considering several image reformations of the inner ear to detect the critical cochlear co-ordinates (e.g., round window, modiolar axis);
- Rater’s freedom from expectations regarding the electrode position;
- Performance benchmark (target performance level) with continuous feedback of the rater’s results; feedback when rater exceeds control limits;
- Awareness of current evidence in cochlear-implant-imaging evaluation (recurring feedback, constructive criticism, suggestions and support);
- Masking of relevant case information that allows conclusions about the electrode position, e.g. surgical report.

**Fig. 1:**
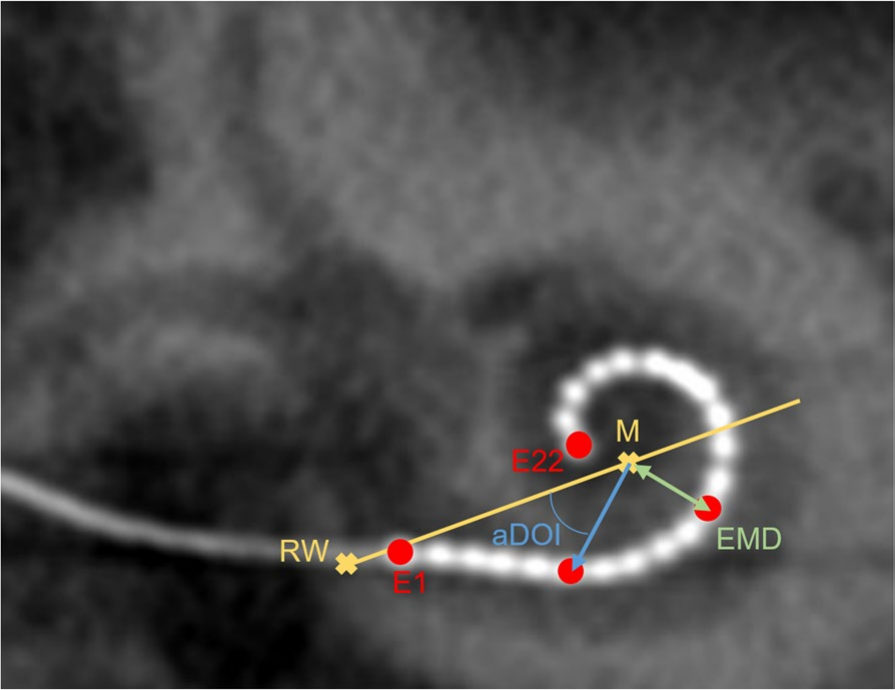
Schematic illustration of the electrode-to-modiolus distance (EMD) and angular depth of insertion (aDOI) to be measured. Applying the cochlear view, EMD was measured from the center of the modiolus “M” (helicotrema) to the center of an electrode contact “E”. A line from the round window “RW” through the modiolus to the lateral wall of the cochlear was the reference for measuring aDOI

Following the idea of linear sequential unmasking (LSU) in nuclear physics [8] and forensics [9, 10], our clinical workflow in assessing the CI electrode position (EMD and aDOI measurements) was sequenced linearly as follows:

1. Fitting a section plane to the basal cochlear turn within the sagittal plane;
2. On the basis of this plane, reconstruction of the “cochlear view” (a two-dimensional cross-sectional image perpendicular to the modiolus and coplanar with the basal turn of the cochlea [11–15]);
3. Optimizing the visualization of the electrode contacts by image-filtering and adjusting the image contrast;
4. Applying a universal co-ordinate system with the helicotrema as center of the modiolus [12];
5. Detecting required landmarks (helicotrema, round window; Fig. 1);
6. Measuring the EMD from the center of the modiolus (helicotrema) to the center of each of the electrode contacts E1, E6, E11, E16 and E22 [16];
7. Defining a line from the round window through the modiolus to the lateral wall of the cochlear as reference for measuring the aDOI;
8. Measuring the aDOI at electrode contacts E1 and E22. The round window was set as zero degree angle [12].

Each of these steps was presented (unmasked) as a learning goal to the student in a sequential manner; irrelevant case information was masked as far as possible.

### Design and setting of the study

The performance of the student during training (repeated feedback/measurement series) was compared with the target performance level achieved by a single senior rater. A prospective experimental study design was chosen. The study was located at a tertiary referral medical center with a cochlear-implant program.

### Subjects

Imaging analysis was done within a clinical training dataset (*N* = 30) consisting two (repeated) EMD and aDOI measurement series of the senior rater. Subjects within this training dataset underwent cochlear implantation with a Nucleus® CI532 Slim Modiolar electrode (*N* = 20) or CI512 Contour Advance electrode (*N* = 10). Median age at implantation was 48 years (interquartile range, IQR: 30 years) and median preoperative PTA4AC was 96 dB (IQR 18 dB). PTA4_AC_ indicated air-conduction pure-tone average threshold (0.5, 1, 2, 4 kHz) measured monaurally with circumaural headphones. Further demographic and CI-related characteristics of the subjects are given in Table 1. All subjects met the following inclusion criteria: round-window approach for intracochlear electrode insertion; absence of tip-foldovers and buckles of the electrode; absence of repeated insertions of the electrode; no anatomical abnormalities of the cochlear and auditory nerve. Furthermore, only tomography results with adequate image quality were analyzed, reducing system-related errors in distance and aDOI measurements. Adequate image quality was defined as the absence of artifacts due to motion and/or beam hardening.

**Table 1:** Demographic, CI-related and imaging characteristics of the subjects

Subject number	Sex	Ear implanted	Etiology of unilateral hearing loss	Implant type	Imaging type	Image matrix	Image voxel size (mm^3^)
1	F	R	Unknown	CI512	CT	1280 × 1280	0.125 × 0.125 × 0.125
2	M	R	Otosclerosis	CI512	CT	800 × 600	0.2 × 0.2 × 0.2
3	M	R	Meniere’s disease	CI512	CT	640 × 640	
4	F	R	Sudden hearing loss	CI512	CT	1280 × 1280	0.125 × 0.125 × 0.125
5	F	L	Unknown	CI512	CT	800 × 600	0.2 × 0.2 × 0.2
6	F	L	Familial	CI512	CT	1280 × 1280	0.125 × 0.125 × 0.125
7	M	R	Meniere’s disease	CI512	CT	1280 × 1280	0.125 × 0.125 × 0.125
8	M	R	Unknown	CI512	CT	1280 × 1280	0.125 × 0.125 × 0.125
9	M	L	Unknown	CI512	CT	1280 × 1280	0.125 × 0.125 × 0.125
10	F	R	Infection	CI512	CT	1280 × 1280	0.125 × 0.125 × 0.125
11	M	R	Unknown	CI512	CT	1280 × 1280	0.125 × 0.125 × 0.125
12	F	R	Unknown	CI512	CT	1280 × 1280	0.125 × 0.125 × 0.125
13	M	L	Familial	CI532	DVT	800 × 600	0.2 × 0.2 × 0.2
14	M	R	Infection	CI532	DVT	800 × 600	0.2 × 0.2 × 0.2
15	F	L	Infection	CI532	DVT	640 × 640	0.25 × 0.25 × 0.25
16	F	R	Infection	CI532	DVT	640 × 640	0.25 × 0.25 × 0.25
17	F	L	Sudden hearing loss	CI532	DVT	800 × 600	0.2 × 0.2 × 0.2
18	M	R	Infection	CI532	DVT	640 × 640	0.25 × 0.25 × 0.25
19	M	L	Sudden hearing loss	CI532	DVT	640 × 640	0.25 × 0.25 × 0.25
20	F	L	Familial	CI532	DVT	640 × 640	0.25 × 0.25 × 0.25
21	F	L	Syndromal	CI532	DVT	640 × 640	0.25 × 0.25 × 0.25
22	M	L	Sudden hearing loss	CI532	DVT	640 × 640	0.25 × 0.25 × 0.25
23	F	R	Unknown	CI532	DVT	800 × 600	0.2 × 0.2 × 0.2
24	F	L	Unknown	CI532	DVT	640 × 640	0.25 × 0.25 × 0.25
25	M	L	Familial	CI532	DVT	640 × 640	0.25 × 0.25 × 0.25
26	M	R	Sudden hearing loss	CI532	DVT	640 × 640	0.25 × 0.25 × 0.25
27	M	L	Unknown	CI532	DVT	640 × 640	0.25 × 0.25 × 0.25
28	M	R	Unknown	CI532	DVT	640 × 640	0.25 × 0.25 × 0.25
29	F	R	Ototoxic	CI532	DVT	640 × 640	0.25 × 0.25 × 0.25
30	F	L	Sudden hearing loss	CI532	DVT	800 × 600	0.2 × 0.2 × 0.2

### Imaging analysis

Imaging was performed during the first week after cochlear implantation as part of our clinical routine. Within the training data set, computer tomography (CT) was used for all CI512 implants and digital volume tomography (DVT) was used for all CI532 implants. Imaging parameters were consistent with respect to an imaging voltage of 70–120 kV, a tube current of 1.1–5 mA/frame with pulsed X-ray emission and an exposure time of 7 s. Resolution and isotropic voxel size are given in Table 1. EMD and aDOI values were extracted from the “cochlear view” (Fig. 1) as previously described. In order to avoid confounding bias, the influence of the type of imaging and the resolution (matrix and voxel size) on the EMD/aDOI interrater differences under investigation was analyzed. For this, statistical tests mentioned in the following section were used to compare the central tendencies of two or more samples.

The software “KaVo eXam Vision” (KaVo Dental GmbH) was available for reconstructing the cochlear view and measuring the distances directly. Each cochlear-view image was further converted to JPEG format for measurement of aDOI using the software “ImageJ” (National Institutes of Health, Bethesda, MD). The lines drawn in this sectional view (EMD) served as the basis for the angle measurements.

### Data analysis

All statistical analyses were performed using the MATLAB™ software (The MathWorks, Inc., Natick, Massachusetts). The measurable variables are EMD and aDOI interrater differences (single senior rater – single student rater) in comparison of the senior’s intrarater differences (test–retest of each image by the single rater). Shewhart charts were used to check whether interrater differences appeared to be within the target range (interpercentile range of the senior rater’s intrarater differences). The interpercentile range (IPR) was defined as the distance between the 0.1th and 99.9th percentiles (P0.1 and P99.9). Since the data were not normally distributed, these percentiles were used here as control limits equivalent to ±3 standard deviation as originally defined by Shewhart [17]. Thus, results by the student are “out of control” if interrater differences exceed the IPR of the senior rater’s intrarater differences.

The Shapiro–Wilk test was applied for testing whether the data were normally distributed. Since normal distribution did not apply to all variables, the Wilcoxon test was used to compare the central tendencies of two samples, and Friedman’s test was used for comparing the central tendencies of several samples. Multiple-comparison post-hoc corrections using Dunn’s test were applied to determine which samples differed from each other. The Brown–Forsythe test was applied to compare the variance of two samples. Statistical significance was defined as *p* < 0.05.

Intrarater reliability was analyzed by intraclass correlation and Bland–Altman analysis. Intraclass correlation coefficient (ICC) was also used to calculate the strength of agreement between the two raters. Following the convention for intraclass correlation by McGraw and Wong [18], a 2-way mixed-effects model, multiple raters/measurements type with absolute agreement definition was chosen.

## Results

### Analysis of target performance

Intrarater reliability was calculated as the difference from two EMD and aDOI measurement series (test–retest) made by a single senior rater served as target performance level for the student. To present results clearly, EMD was summarized across all five electrode contacts measured (EMD_5_). Neither central tendencies (*p* = 0.77) nor variances (*p* = 0.24) of the senior’s intrarater EMD_5_ differences differed statistically among these five electrode contacts. However, statistically significant differences were found in the variances of aDOI measurement series differences between contacts E1 and E22 (*p* < 0.001); therefore, these results were further analyzed, separately, for each of those contacts.

Sufficient intrarater reliability of the senior rater was ensured by means of intraclass correlation and Bland–Altman analysis. For EMD_5_, the intraclass correlation coefficient ranged from 0.98 to 0.99, corresponding to an excellent intrarater reliability [19]. Intrarater reliability ranged from good to excellent for aDOI at electrode contacts E1 and E22, with ICC ranging from 0.82 to 0.96 and from 0.91 to 0.98, respectively.

**Fig. 2:**
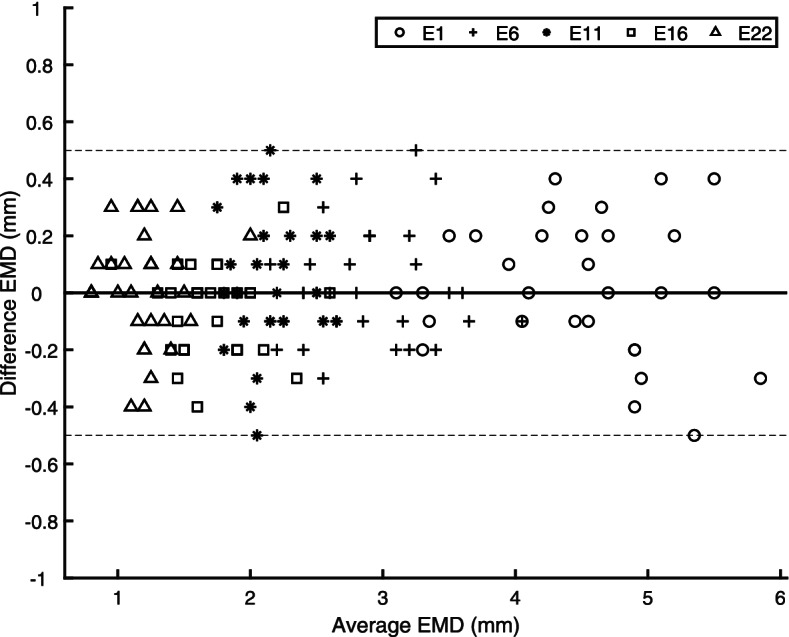
Differences versus averages of two EMD measurement series made by a senior rater (*N* = 30). Applying the cochlear view, EMD was measured from the center of the modiolus (helicotrema) to the center of each of the electrode contacts E1, E6, E11, E16 and E22. A bold solid line indicates the median value (bias) of the senior rater’s intrarater differences; both dotted lines cover the 0.1th to 99.9th interpercentile range

**Fig. 3:**
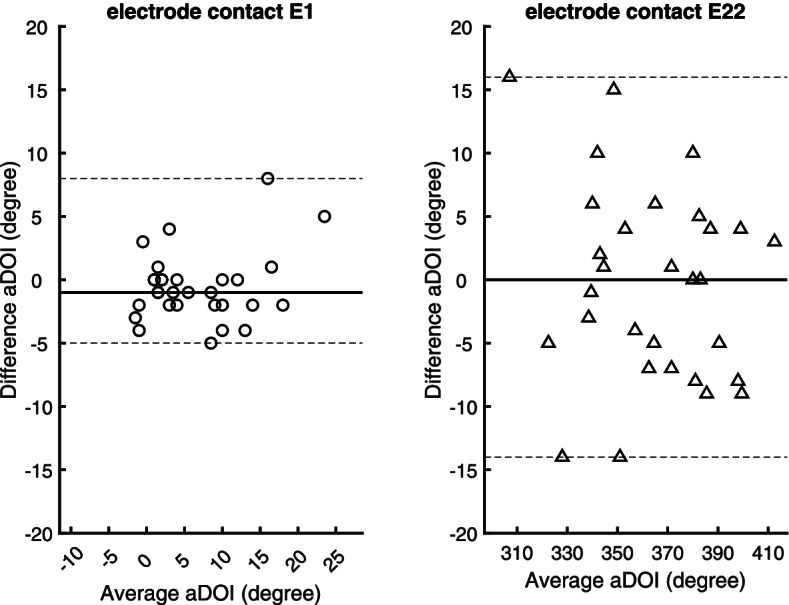
Differences versus averages of two aDOI measurement series made by a senior rater (*N* = 30). Applying the cochlear view, a line from the round window through the modiolus to the lateral wall of the cochlear was the reference for measuring the aDOI at electrode contacts E1 (left panel) and E22 (right panel). A bold solid line indicates the median value (bias) of the senior rater’s intrarater differences; both dotted lines cover the 0.1th to 99.9th interpercentile range

As the intraclass correlation coefficient is expected to be high in repeated measurements by a single rater, intraclass correlation was supplemented by a Bland–Altman analysis. For EMD_5_, the median difference (median bias) was 0 mm for the senior rater (Fig. 2; percentiles P0.1/P99.9 = ± 0.5 mm). Analyzing the aDOI at electrode contacts E1 and E22 revealed a median difference of − 1° and 0°, respectively (Fig. 3). The percentiles P0.1 and P99.9 were found to be − 5° and 8° for E1 and − 14° and 16° for E22. Median bias and spread did not vary with the average of the two measurement series, for all three variables (EMD_5_, aDOI at E1, aDOI at E22).

### Learning curves of the student rater

**Fig. 4:**
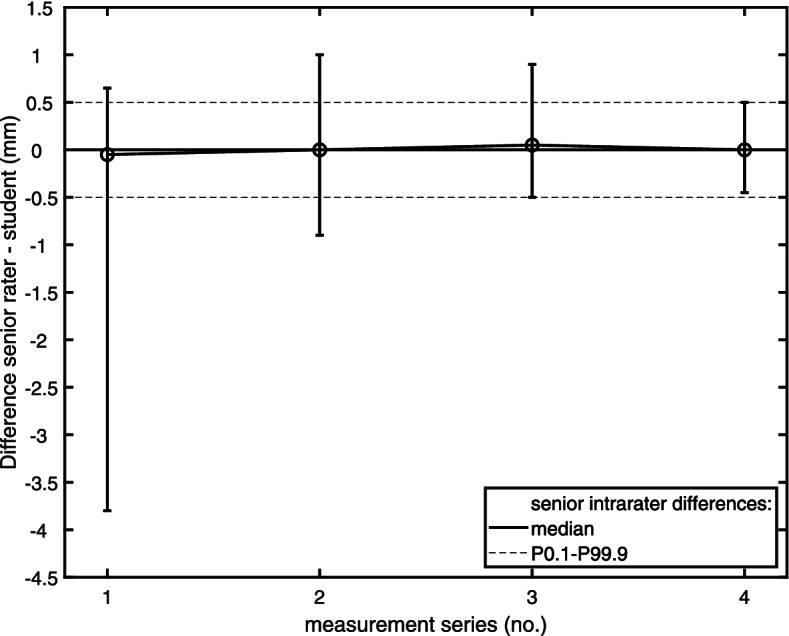
Shewhart chart of a student measuring EMD (*N* = 30). In each measurement series, interrater EMD differences (average of two series by a senior rater – student) are plotted. Error bars cover the 0.1th to 99.9th interpercentile range (IPR) of these interrater differences, according to the control limits given by the senior rater’s intrarater IPR (dotted lines). A bold solid line indicates the median value of the senior rater’s intrarater differences. Median interrater differences are linked across the four measurement series by a thin solid line

The Shewhart control chart for EMD_5_ is shown in Fig. 4. In each measurement series by the student, interrater EMD_5_ differences (average of two series by the senior rater relative to the student) were plotted. Error bars cover the IPR of these interrater differences, according to the control limits given by the (intrarater) IPR of the senior rater. Interrater differences did not differ from senior intrarater differences in any of the four measurement series when central tendencies were compared (*p* = 0.46). However, a learning effect was observed with respect to the scatter of the interrater differences. This scatter became smaller with successive measurement series; the target performance level was achieved in the 4th series. The intraclass correlation coefficient for the interrater IPR of this final series and the intrarater IPR of the senior rater ranged from 0.98 to 0.99.

**Fig. 5:**
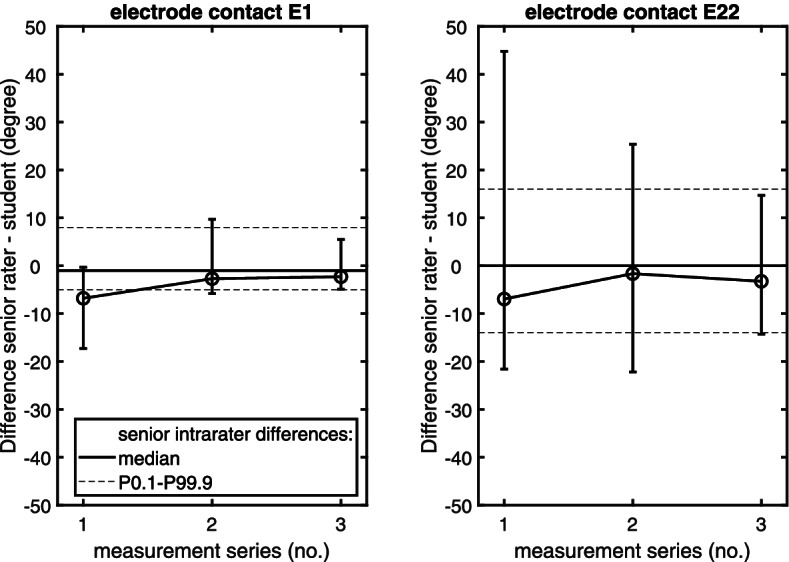
Shewhart chart of the student measuring the aDOI at electrode contact E1 (left panel) and E22 (right panel). In each of the student’s measurement series, interrater EMD differences (average of two series by a senior rater – student; *N* = 30) are plotted. Error bars show the 0.1th to 99.9th interpercentile range (IPR) of these interrater differences, according to the control limits given by the intrarater IPR of the senior rater (dotted lines). A bold solid line indicates the median value of the senior rater’s intrarater differences. Median interrater differences are linked across the three measurement series by a thin solid line

Analysis of interrater aDOI differences at electrode contacts E1 and E22 also showed a reduction in their scatter along the measurement series (Fig. 5). Compared with the EMD_5_ results, however, an interrater IPR less than or equal to the senior rater’s intrarater IPR was already achieved with the third measurement series. Intraclass correlation coefficient for the interrater IPR of this final series and the intrarater IPR of the senior rater ranged from 0.71 to 0.93 (E1) and from 0.88 to 0.97 (E22).

In both electrode types (CI512, CI532) and electrode contacts (E1, E22) tested, Friedman’s test indicated significant differences between the four data samples (interrater differences in measurement series 1 to 3; senior intrarater differences; *p* < 0.05). Post-hoc analysis revealed statistically significant differences between the student’s first series and all others (for E1) and between this first series and the senior’s intrarater differences (E22; Dunn’s test, *p* < 0.05). Thus, in contrast to the EMD_5_ findings, the aDOI results in the first measurement series differed from the target range not only in scatter, but also in central trend.

### Confounding analysis

We identified the type of implant and imaging technique, and also image resolution and voxel size, as potential confounding variables in measuring the EMD and aDOI. Since CT was used for all CI512 implants and DVT was used for all CI532 implants, confounder analysis for type was done only once (CI512/CT versus CI532/DVT). Similarly for the image resolution, where the matrix and voxel size were linked: resolution low (640 × 640 and 0.25 mm), medium (800 × 600 and 0.2 mm) and high (1280 × 1280 and 0.125 mm). For both confounding variables, we investigated whether there were statistically significant differences in interrater differences between the characteristics.

Considering the EMD_5_, no significant differences were found for type (*p* = 0.99) or resolution (*p* = 0.86).

In contrast, aDOI measurements at electrode contact E22 revealed a significant difference for type (*p* < 0.01). Interrater differences for C512/CT measurements were lower than for CI532/DVT (median − 9° versus 1°, IPR 28° versus 17°) – i.e., the student reported greater aDOI values in CI512/CT than the senior rater. However, at electrode contact E1, interrater aDOI differences did not differ significantly between the two types (*p* = 0.06).

Considering the image resolution for interrater aDOI differences at electrode contacts E1 and E22, Friedman’s test indicated significant differences between the resolution categories (low/medium/high; *p* < 0.05). Post-hoc analyses revealed significantly different mean ranks for the low- and high-resolution samples (Dunn’s test, *p* < 0.05). The median of the aDOI differences was shifted to more negative values for the low resolution (E1: –4° with IPR 7 degree; E22: –8°, IPR 17°) than for the high resolution (E1: 0°, IPR 10°; E22: 3°, IPR 28°). This resulted from larger measured aDOI values of the student in images with low resolution compared with those of high resolution.

Nevertheless, confounding effects of implant/imaging type and resolution on aDOI were within the intrarater IPR of the senior rater, so no further allowance was made for them.

## Discussion

Even with the introduction of automated tools for analyzing post-operative CI images [20–24], the manual evaluation of CI electrode position is still of general interest in clinical practice. However, findings can be missing or misinterpreted. For such diagnostic errors, sources of cognitive bias are of great importance, and can influence both experienced and inexperienced raters [4, 5]. Therefore, clinical quality assurance should both address the training of inexperienced raters and monitor continuously the performance of experienced raters.

To the best of our knowledge, no quality–assurance methods exist to train inexperienced raters in clinical evaluation of the CI electrode position. In this study, we introduce an approach that enabled a medical student to measure electrode-to-modiolus distances and angular insertion depth, both at the performance level of an experienced rater. This training approach requires time, staff and material resources, but is in line with international demands on clinical quality assurance [25, 26].

Moreover, the performance data within this training approach can be used as a benchmark to establish an ongoing clinical quality control. Benchmarking diagnostic performance is a key corrective strategy for reducing cognitive bias and has already been described as useful for quality control in radiology [1, 27, 28]. This seems reasonable, as raters are apparently vulnerable to heuristic failures, even when their learning process is complete [5].

Owing to the simplicity of this process, especially with regard to the small number of measurement series made by the student, we decided to use a Shewhart chart (an established statistical control tool in economics) to monitor training success [17]. However, Shewhart analysis is inefficient in detecting small changes (up to ±2 standard deviations) of the variable being monitored [29]. Thus, further clinical benchmarking could combine a Shewhart control chart with an analysis of cumulative sums (CUSUM) [30, 31]. A CUSUM chart is more efficient in detecting small changes in the process mean, as the control limits are more complex in design than with Shewhart (mostly V-mask design versus constant terms). Although the design of a CUSUM control chart may be complex, it has already been shown useful in evaluating competence in clinical procedures [32–35].

Use of even reference (target) data obtained by a single senior rater may limit their representativeness compared with a benchmark with data from several experienced raters. However, this work was to test only the general procedure, as a proof of concept. The measurable variables (EMD and aDOI differences between student and senior rater) were therefore compared with the latter’s intrarater differences for independence of the EMD and aDOI magnitude: the senior rater’s intrarater reliability in measuring EMD and aDOI was conformed by both correlations and by a Bland–Altman analysis. Nevertheless, our results simply demonstrate successful learning curves in measuring EMD and aDOI for a single student compared with a single senior rater.

For the last measurement series of both EMD and aDOI, the median was close to zero for intrarater differences of the senior rater as well as for interrater differences. However, we also analyzed the scatter. Following common practice in Shewhart analysis, this scatter covered 99.8% of data (± 3 standard deviations as defined by Shewhart [17]). Intrarater EMD differences (± 0.5 mm), on the one hand, were greater than the resolution used here (≤ 0.25 mm) and are thus clinically relevant. Nonetheless, the literature lacks comparative data for intrarater and interrater EMD differences. In several studies electrode-to-modiolus distances in cochlear implants was measured [16, 36–44]. However, information on intrarater or interrater differences is omitted, or simply the agreement between two raters is calculated; this was found to be similar to our results (ICC for EMD between 0.77 and 1 [40]).

In contrast to EMD, Fernandes et al. (2018) [45] measured aDOI and found a mean difference of − 0.9° with a standard deviation of 43°. Since interrater differences were calculated for several raters, a greater scatter seems reasonable compared with the intrarater dispersion (up to 16°) for the single rater in the present study. Svrakic et al. (2015) [46] observed average intrarater aDOI differences within 10°, but provide no data on dispersion. Furthermore, without reporting intrarater or interrater differences, Escudé et al. (2006) [47] stated that aDOI measurements were performed by highly experienced radiologists. As cognitive bias can lead to diagnostic errors even for experienced raters, we suggest always quantifying the error made by the rater(s) when interpreting measurements in CI imaging. The central tendency and the scatter of intrarater/interrater differences should be indicated.

## Conclusions

Evaluating CI electrode position by subjective image-processing may be affected by cognitive bias. The training of inexperienced raters marks the first step toward quality-assured evaluation of CI images. By the training approach presented here, a single medical student became able to measure electrode-to-modiolus distances and insertion-depth angles at the performance level of a single senior rater. This approach should allow systematic teaching of knowledge and skills while applying corrective strategies to reduce faulty data-gathering and synthesis. Shewhart control charts can be used to monitor individual learning curves. Benchmarking the performance as a key corrective strategy can provide a basis for ongoing clinical quality assurance in evaluating the CI electrode position. This should also include quantifying the error made by rater(s) in interpreting measurements in CI imaging.

## Supplementary Information

**Additional file 1.** The raw aDOI and EMD data collected from imaging for analysis. M1 indicates measurement series no. 1, M2 series no. 2, etc.

## Data Availability

All data obtained and analyzed during this study are included in this published article and its supplementary information files.

## Abbreviations

EMD: Electrode-to-Modiolus Distance
aDOI: Angular Depth Of Insertion
CI: Cochlear Implant
CT: Computer Tomography
DVT: Digital Volume Tomography
LSU: Linear Sequential Unmasking
IQR: Interquartile Range
PTA4AC: Air-Conduction Pure-Tone Average threshold (0.5, 1, 2, 4 kHz)
IPR: Interpercentile Range
ICC: Intraclass Correlation Coefficient
CUSUM: Analysis of Cumulative Sums
